# Soil respiration patterns and rates at three Taiwanese forest plantations: dependence on elevation, temperature, precipitation, and litterfall

**DOI:** 10.1186/s40529-017-0205-7

**Published:** 2017-11-15

**Authors:** Yu-Hsuan Huang, Chih-Yu Hung, I-Rhy Lin, Tomonori Kume, Oleg V. Menyailo, Chih-Hsin Cheng

**Affiliations:** 1 School of Forestry and Resource Conservation, National Taiwan University, Taipei, 106 Taiwan; 2 Institute of Forest Research RAI SR, Krasnoyarsk, 660036 Russia

**Keywords:** Soil respiration, Elevation, Soil temperature, Soil water content, Litterfall, Temperature sensitivity

## Abstract

**Background:**

Soil respiration contributes to a large quantity of carbon emissions in the forest ecosystem. In this study, the soil respiration rates at three Taiwanese forest plantations (two lowland and one mid-elevation) were investigated. We aimed to determine how soil respiration varies between lowland and mid-elevation forest plantations and identify the relative importance of biotic and abiotic factors affecting soil respiration.

**Results:**

The results showed that the temporal patterns of soil respiration rates were mainly influenced by soil temperature and soil water content, and a combined soil temperature and soil water content model explained 54–80% of the variation. However, these two factors affected soil respiration differently. Soil temperature positively contributed to soil respiration, but a bidirectional relationship between soil respiration and soil water content was revealed. Higher soil moisture content resulted in higher soil respiration rates at the lowland plantations but led to adverse effects at the mid-elevation plantation. The annual soil respiration rates were estimated as 14.3–20.0 Mg C ha^−1^ year^−1^ at the lowland plantations and 7.0–12.2 Mg C ha^−1^ year^−1^ at the mid-elevation plantation. When assembled with the findings of previous studies, the annual soil respiration rates increased with the mean annual temperature and litterfall but decreased with elevation and the mean annual precipitation. A conceptual model of the biotic and abiotic factors affecting the spatial and temporal patterns of the soil respiration rate was developed. Three determinant factors were proposed: (i) elevation, (ii) stand characteristics, and (iii) soil temperature and soil moisture.

**Conclusion:**

The results indicated that changes in temperature and precipitation significantly affect soil respiration. Because of the high variability of soil respiration, more studies and data syntheses are required to accurately predict soil respiration in Taiwanese forests.

## Introduction

Soil respiration, which refers to the efflux of carbon dioxide from the soil surface to the atmosphere, is a crucial component of carbon cycling. It is the primary pathway through which carbon moves from the ecosystem into the atmosphere, and it strongly affects the carbon balance and turnover (Raich and Schlesinger 1992; Davidson and Janssens 2006; Bond-Lamberty and Thomson 2010). In the forest ecosystem, the soil contributes to 60–80% of total ecosystem respiration (Law et al. 1999; Janssens et al. 2001). Understanding the rate of carbon release from the soil and its seasonal patterns will lead to a comprehensive understanding of how to tackle regional and global climate change.

Soil respiration is produced mainly by plant roots and soil organisms. The soil respiration rates are largely dependent on soil temperature and soil water content. Most related studies have shown that soil respiration is positively correlated with soil temperature (Davidson et al. 1998), and that it can be modified or even impeded by soil water content under very wet or very dry conditions (Davidson et al. 2000; Raich and Schlesinger 1992). The effects of soil temperature and soil water content on soil respiration have been suggested to be interactive, rather than individual (Raich and Schlesinger 1992; Davidson et al. 1998; Reichstein et al. 2003; Saiz et al. 2006). Global meta-analysis data indicated that soil respiration is significantly correlated with mean annual temperature (MAT) and mean annual precipitation (MAP) (Raich and Schlesinger 1992) and possibly also with three variables—MAT, MAP, and vegetation productivity (Raich and Schlesinger 1992; Reichstein et al. 2003; Bond-Lamberty and Thomson 2010). However, the majority of these studies were conducted in temperate ecosystems; fewer measurements have been taken in the tropics and subtropics. Some measurements taken in the tropics and subtropics demonstrated a high deviation between measurements and estimations made using global regression models, with the differences ranging from − 73 to + 294% (Hashimoto et al. 2004; Chang et al. 2008; Katayama et al. 2009; Tan et al. 2013). These statistics exemplify the inherent variability of soil respiration in the tropics and subtropics; thus, soil resperation in these regions should be further investigated.

Because of the high cost of measurement instruments and the complex landscape, soil respiration has not been extensively investigated in Taiwan (Chang et al. 2008; Kao and Chang 2009; Hsieh et al. 2016). Therefore, soil respiration in different areas and its seasonal dynamics are not widely understood. In Taiwan, the temperature decreases with elevation, with a lapse rate of − 5.3 °C km^−1^. However, precipitation increases with elevation, with mountainous areas and lowlands receiving 2000–3500 and 1500–2000 mm of annual rainfall, respectively (Lee et al. 2004). Such climatic differences associated with their influences over primary production may have significant impacts on soil respiration on spatial and temporal scales. The mechanisms underlying the effects of these factors on soil respiration must be determined to enable the assessment of the interaction between climate and soil respiration and the processes involved in the interaction. Moreover, measuring the soil respiration rates in Taiwan would ensure the completeness of the global database, particularly for databases for tropical and subtropical ecosystems (Raich and Schlesinger 1992; Bond-Lamberty and Thomson 2010).

In this study, soil respiration at three forest plantations (two lowland and one mid-elevation) was investigated throughout an annual cycle. Soil temperature, soil water content, and litterfall production were also determined at each plantation. We investigated the soil respiration rate at each plantation and compared the rate among the plantations. Our objectives were as follows: (1) to determine how soil respiration varies between lowland and mid-elevation forest plantations, (2) to analyze the relative importance of soil temperature and soil moisture for temporal soil respiration patterns at the three studied plantations, and (3) to assess which factors are crucial for controlling the annual soil respiration rate across the plantations.

## Materials and methods

### Study sites

The first plantation in this study is located in the lowland plain in Rende District, Tainan (TN; 22°56′N, 120°16′E). This plantation was originally an orchard but had been abandoned for more than 15 years. It is now used as a representative plantation for predicting potential growth in current cropland afforestation areas because no such old plantation exists in Taiwan (Lin et al. 2011; Cheng et al. 2016a). Two stands, TN1 and TN2, were evaluated. TN1 is an abandoned lychee (*Litchi chinensis*) plantation with a stand age of 33 years. TN2 is an abandoned wax apple (*Eugenia javanica*) plantation with a stand age of 25 years. Because of the long-term abandonment, understory vegetation such as *Macaranga tanarius*, *Murraya paniculata*, *Celtis sinensis*, *Panicum maximum*, and *Alpinia speciose* grows rigorously at both stands.

The second plantation is located in the Datu tableland, Taichung (TC; 24°17′N, 120°35′E). This plantation is an *Acacia confusa* plantation situated at an elevation of 150 m above sea level. Two adjacent stands, TC1 and TC2, were studied. Both stands have the same *Acacia* overstory but differ in their understory vegetation because of the influence of fire disturbance and invasive grass. TC1 has not been disturbed by fire, and the dominant understory species are *Lantana camara*, *Zanthoxylum nitidum*, *Mallotus repandus*, and *Glochidion rubrum*. However, TC2 has been slightly influenced by ground fire, and the understory vegetation (more than 90% coverage) has been replaced by grass (*Panicum maximum*) (Cheng et al. 2013a).

The third plantation is located in mid-elevation montane areas in Xitou, Nantou (NT; 23°40′N, 120°46′E). The plantation is a Japanese cedar (*Cryptomeria japonica*) plantation situated at an elevation of approximately 1200 m above sea level. Three stands, NT1, NT2, and NT3, were studied. The stand ages of which are 90, 63, and 40 years, respectively, at the time of this study. In these three stands, the mean diameter at breast height (DBH), basal area, and living tree biomass carbon stocks increase with stand age. By contrast, tree density decreases with stand age (Cheng et al. 2013b) (Table 1).

**Table 1:** Stand characteristics and soil properties from surface level to a depth of 10 cm in the selected stands at TN, TC, and NT plantations in Taiwan

Site	Elevation (m)	MAT (°C)	MAP (mm)	Dominant vegetation	Trees (multi-trunks) density (No. ha^−1^)	Mean trunk DBH (cm)	Basal area (m^2^ ha^−1^)	Canopy height (m)	LAI^a^	Litterfall (Mg C ha^−1^ year^−1^)	Soil texture	pH	Soil organic carbon (%)
TN1	14	24.3	1698	*L. chinensis*	560 (1566) ± 134 (492)	16.7 ± 0.6	40.7 ± 3.2	11.8	4.4	5.4 ± 0.7	Loam	4.7 ± 0.1	2.2 ± 0.2
TN2	13			*E. javanica*	321 (2821) ± 91(855)	12.8 ± 0.7	68.5 ± 12.1	10.6	3.9	5.8 ± 1.1	Sandy loam	4.8 ± 0.1	1.5 ± 0.1
TC1	110	23.0	1347	*A. confusa*	2360 ± 433	10.0 ± 0.9	21.7 ± 3.8	9.3	3.3	3.8 ± 0.2	Loam	3.9 ± 0.1	4.9 ± 0.4
TC2	110			*A. confusa*	2533 ± 985	9.9 ± 2.7	21.0 ± 3.6	8.4	3.3	3.8 ± 0.2	Loam	3.8 ± 0.0	4.5 ± 0.1
NT1	1150	16.6	2635	*C. japonica*	408 ± 22	48.6 ± 1.2	77.9 ± 5.9	29.0	4.0	2.0 ± 0.4	Loam	4.0 ± 0.3	17.2 ± 2.8
NT2	1250			*C. japonica*	617 ± 8	35.3 ± 1.0	63.7 ± 4.4	26.0	3.4	1.9 ± 0.2	Sandy loam	5.8 ± 0.1	3.4 ± 0.8
NT3	1370			*C. japonica*	1358 ± 162	23.1 ± 0.4	59.7 ± 4.0	20.8	4.5	3.6 ± 0.8	Loam	4.5 ± 0.1	4.3 ± 0.8

Climate data referred to Central Weather Bureau, Taiwan; Stand structure referred to Cheng et al. (2013a, b, 2016b) and Lin et al. (2011)

*MAT* mean annual temperature, *MAP* mean annual precipitation

^a^ *LAI* leaf area index, was determined using a LI-COR 2200 (LI-COR Comp., Lincoln, Nebraska, USA)

Elevation, MAT, and MAP at the three study plantations are shown in Table 1. MAT is strongly affected by elevation and ranges from 16.6 °C at NT to 25.0 °C at TN. Elevation also exerts a significantly orographic influence on precipitation. A higher MAP was found at the mid-elevation NT plantation (2635 mm) than at the lowland plantations at TN (1698 mm) and TC (1347 mm). The climatic conditions at both TN and TC correspond to tropical and subtropical moist forests, and the climatic condition at NT is classified as a montane wet subtropical forest.

Previously studied stand characteristics and soil properties are shown in Table 1 (Lin et al. 2011; Cheng et al. 2013a, b, 2016b). Generally, all selected stands were closed canopy with leaf area indices between 3.3 and 4.5. The canopy height ranged between 8.4 and 29.0 m, and the mean stem DBH ranged between 9.9 and 48.6 cm. The soil textures were loam and sandy loam. Stand productivity was evaluated based on aboveground litterfall production, which has been correlated to the total net primary production and has been used to represent the labile input available to decomposition under a forest ecosystem in previous studies (Bond-Lamberty and Thomson 2010). Annual aboveground litterfall production over study stands ranged between 1.9 and 5.8 Mg C ha^−1^ (Table 1). Higher litterfall production was found at TC and TN than at NT.

**Fig. 1:**
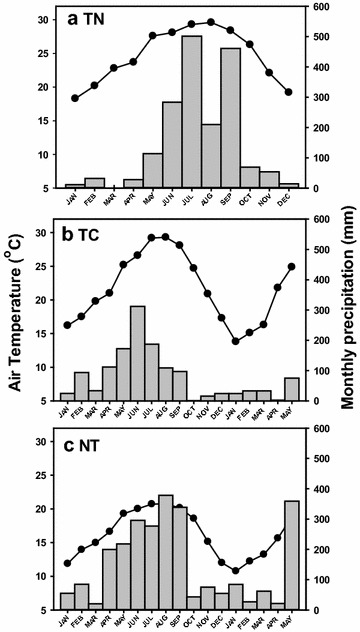
Monthly mean air temperature (°C, black circle) and precipitation (mm, gray bar) from January 2010 to December 2010 at TN plantation and from January 2010 to May 2011 at TC and NT plantations

In 2010, the mean air temperature was 24.6, 23.0, and 17.0 °C, and the total precipitation was 1778, 1182, and 2041 mm at TN, TC, and NT, respectively (Fig. 1). The annual air temperature and precipitation at TN and TC in 2010 were consistent with their long-term climate regimes, but lower precipitation was found at NT (2041 vs. 2500 mm) because of lower precipitation in summer. Generally, a hot and humid season from April to September and a cold and dry season from October to March can be observed at all three plantations.

### Soil respiration

Soil respiration was measured using a Li-6400 with a Li-6400-09 soil chamber (LI-COR Inc., Lincoln, Nebraska, USA). Measurements were taken using polyvinyl chloride (PVC) collars that were 11 cm in diameter and 7 cm in height. The collars were installed on the soil surface at a depth of 5 cm to prevent erosion from heavy rainfall and enable the growth of fine roots. Six PVC collars (duplicates from three 20 × 20-m^2^ plots) were applied at each stand. The collars were installed 2 months before the first measurement and remained in place throughout the measurement period. Soil respiration at each collar was calculated as the average of three values generated from three continuous cycles. All chamber measurements were conducted between 08:00 and 14:00 local time to minimize sampling bias introduced by the time of day (Knowles et al. 2015; Hsieh et al. 2016), and measurements were taken monthly from January 2010 to May 2011 at TC and NT and from January 2010 to December 2010 at TN.

The temperature and water content of soil next to each collar were determined during the measurement period. Soil temperature at a depth of 10 cm was measured using the temperature sensor attached to the Li-6400. Soil water content was measured as volumetric soil moisture content by using time domain reflectometry probes (Hydrosense, Campbell Scientific Inc., Utah, USA). In addition, the full spectra of soil temperature and soil water content were recorded. A data logger (HOBO U30, Onset, Massachusetts, USA) connected to a temperature probe (S-TMB-M006, Onset) at a depth of 10 cm and a soil water content probe (S-SMx-M005, Onset) covering a depth of 0–20 cm were used at each stand. Both probes were programmed to measure values at 4-min intervals and store data hourly.

### Modeling temporal soil respiration patterns

To determine whether temporal changes in soil respiration are related to soil temperature and soil water content, the relationships between soil respiration and soil temperature and soil water content were examined using three empirical regression models. The first model involved only soil temperature, and an exponential model was applied (Lloyd and Taylor 1994; Sheng et al. 2010).

1 $${\text{Soil respiration}}\,(\upmu\,{\text{mol CO}}_{ 2} \;{\text{m}}^{ - 2} \;{\text{s}}^{ - 1} ) = {\text{a}} \times { \exp }\;\left( {{\text{b}} \times {\text{T}}} \right)$$

where T is soil temperature (°C) at a depth of 10 cm, and a and b are constants fitted using least squares techniques.

The second model used soil water content as the only indicator variable, and a quadratic equation was applied (Saiz et al. 2006).

2 $${\text{Soil respiration }} = {\text{a}} + {\text{b}} \times \uptheta + {\text{c}} \times \uptheta^{ 2}$$

where θ refers to soil water content (cm^3^ cm^−3^) over a surface at a depth of 20 cm, and a and b are constants fitted using least squares techniques.

In the third model, the soil respiration rate is the function of soil temperature and soil water content (Saiz et al. 2006; Sheng et al. 2010). The model is expressed as follows:

3 $${\text{Soil}}\,{\text{respiration}} = {\text{a}} \times { \exp }\,\left( {{\text{b}} \times {\text{T}}} \right) \times \uptheta^{\text{c}}$$

where T is soil temperature, θ is soil water content, and a, b, and c are the model coefficients.

A Q_10_ function was used to determine the temperature–respiration relationship (Lloyd and Taylor 1994). The equation is expressed as follows:

4 $${\text{Q}}_{ 10} = { \exp }\,\left( { 10 \times {\text{b}}} \right)$$

where b is the model coefficient in Eqs. (1) and (3).

### Data analysis

The regression models were fitted using *R (2.15.2)* to estimate parameters a, b, and c; coefficients of determinations (R^2^); and the root mean squared error (RMSE). The R^2^ and RMSE were used to determine the significance and goodness-of-fit of the models. The soil respiration model was estimated based on the regression model with the highest R^2^ and the lowest RMSE. We applied the continuous measurements of soil temperature and soil moisture from the data logger to the regressive equation to estimate the amount of carbon released through soil respiration. Although the values of the monthly field measurements and data logger were measured using different instruments and locations, their readings were closely correlated (r > 0.98). A linear equation that converted data logger data to field measurements was used before running the regressive equation. The daily soil respiration rate was firstly calculated. The annual soil respiration in 2010 was obtained by calculating the sum of daily estimations (Saiz et al. 2010).

To assess which factors are crucial for controlling the annual soil respiration rates across the plantations, the relationships among annual soil respiration rates, elevation, MAT, and litterfall production were examined. In addition to our study data, data from previous studies that used the same dynamic closed chamber method to measure soil respiration were assembled to generate a more robust relationship (Chang et al. 2008; Kao and Chang 2009; Hsieh et al. 2016).

## Results

### Temporal patterns of soil temperature, soil water content, and soil respiration

**Fig. 2:**
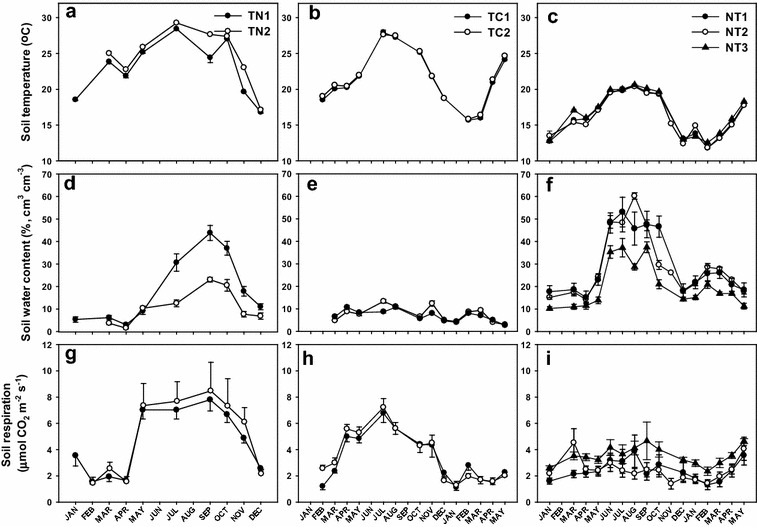
Seasonal patterns of soil temperature (°C), soil water content (%, cm^3^ cm^−3^), and soil respiration rate (μmol CO_2_ m^−2^ s^−1^) at TN (**a**, **d**, **g**), TC (**b**, **e**, **h**), and NT (**c**, **f**, **i**) plantations in Taiwan. Each symbol is the mean of the measurements, and the standard deviation is plotted as error bars

Soil temperature at a depth of 10 cm was low in winter and reached its peak in summer (Fig. 2), ranging between 15 and 30 °C at the lowland TN and TC plantations and between 12 and 20 °C at the mid-elevation NT plantation. The temporal patterns of soil water content were similar to those of soil temperature at all three plantations, namely high in summer and low in winter (Fig. 2). The highest soil water content was observed at NT, reaching > 50% cm^3^ cm^−3^ from June to October because of higher precipitation. The lowest soil water content (< 10% cm^3^ cm^−3^) was observed at TC and TN from December to March because of lower precipitation. According to monthly field measurements, soil water content at TC was below 15% cm^3^ cm^−3^; however, data logger-recorded soil water content ranged between 3 and 34% cm^3^ cm^−3^. The lower values of the monthly field measurements may be due to the placement of the probe at the same soil spot, which might have been desiccated after the measurements were taken. Nevertheless, a linear correlation was observed between the monthly field measurements and data logger data (r = 0.98, p < 0.01).

The seasonality of soil respiration rates at the lowland TC and TN plantations coincided strongly with soil temperature and soil water content (Fig. 2). The soil respiration rate in the hot and humid season was significantly higher than that in the cold and dry season. The soil respiration rates increased from 2 μmol CO_2_ m^−2^ s^−1^ in winter to 8 μmol CO_2_ m^−2^ s^−1^ in summer. However, the soil respiration rate at NT slightly increased from 2 μmol CO_2_ m^−2^ s^−1^ in winter to 3.5 μmol CO_2_ m^−2^ s^−1^ in summer.

### Regression model of soil respiration

The soil temperature univariate model explained approximately 60% of the variations in soil respiration (Table 2). The soil water content model explained 52–82% of the variations at TN and TC but poorly explained those at NT (R^2^ < 34%). The application of a combined soil temperature and soil water content model [Eq. (3)] yielded a higher R^2^ (54–80%) and lower RMSE than did the univariate models. This finding suggested a better representation of the relationship by using both soil temperature and soil water content rather than a single factor. The combined models showed that soil temperature positively contributed to soil respiration at all three plantations. However, the models revealed bidirectional relationships between soil respiration and soil water content. Higher soil moisture content at TN and TC resulted in higher soil respiration rates, whereas higher soil water content at NT led to adverse effects and lower soil respiration rates.

**Table 2:** Estimated parameters of a, b, and c; coefficients of determinations (R^2^); root mean squared error (RMSE); and temperature sensitivity (Q_10_) of regressions for modeling soil respiration at TN, TC, and NT plantations in Taiwan

Model	a	b	c	R^2^	RMSE	Q_10_	a	b	c	R^2^	RMSE	Q_10_
	TN1						TN2					
Temperature	0.592	0.089		0.430	1.965	2.44	0.269	0.120		0.605	1.920	3.31
Water content	1.709	0.246	− 0.003	0.654	1.648		0.053	0.870	− 0.022	0.824	1.400	
Temperature and water content	*0.862*	*0.033*	*0.349*	*0.705*	*1.529*	*1.39*	*0.657*	*0.053*	*0.371*	*0.847*	*1.310*	*1.70*
	TC1						TC2					
Temperature	0.333	0.106		0.611	1.182	2.88	0.374	0.103		0.573	1.359	2.80
Water content	1.073	0.095	0.032	0.565	1.290		0.971	0.214	0.013	0.523	1.506	
Temperature and water content	*0.181*	*0.070*	*0.738*	*0.843*	*0.783*	*2.01*	*0.051*	*0.099*	*1.133*	*0.669*	*1.255*	*2.69*
	NT1						NT2					
Temperature	0.573	0.086		0.676	0.444	2.37	1.347	0.036		0.100	0.826	1.44
Water content	3.864	− 0.138	0.002	0.336	0.663		4.057	− 0.104	0.001	0.085	0.865	
Temperature and water content	*0.744*	*0.128*	− *0.289*	*0.755*	*0.402*	*3.60*	*3.030*	*0.116*	− *0.652*	*0.453*	*0.668*	*3.19*
	NT3											
Temperature	1.394	0.055		0.721	0.376	1.74						
Water content	4.049	− 0.086	0.003	0.278	0.631							
Temperature and water content	*1.515*	*0.065*	− *0.086*	*0.748*	*0.373*	*1.92*						

Italic text represents the best model with the highest R^2^ and lowest RMSE, which was used for estimating annual soil respiration rates

The temperature-dependent Q_10_ values ranged between 2.44 and 3.31 at the lowland TN and TC plantations and between 1.44 and 2.37 at the mid-elevation NT plantation. However, when considering soil water content in Eq. (3), lower Q_10_ values from 1.39 to 2.69 at TN and TC plantations and a higher Q_10_ value from 1.92 to 3.60 at NT plantation were found. This finding suggested that the responses of soil respiration to soil temperature were confounded by soil water content.

### Annual soil respiration rates at different plantations

**Fig. 3:**
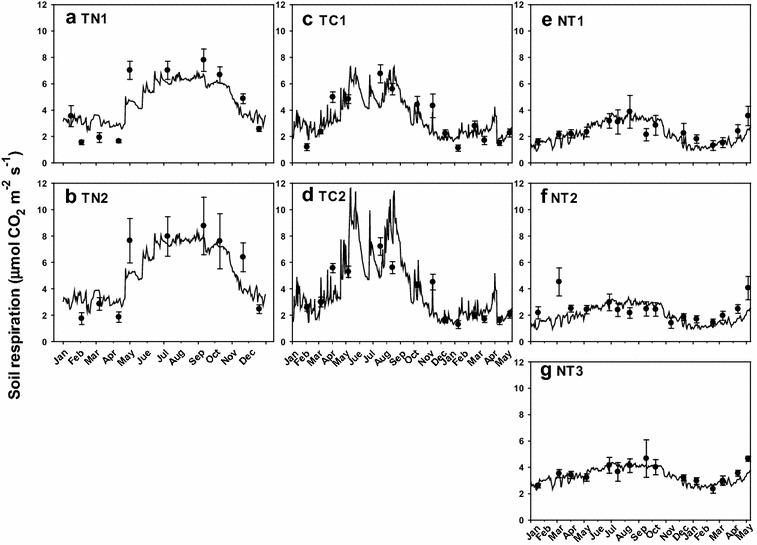
Soil respiration rate (μmol CO_2_ m^−2^ s^−1^) measured in the field (black circles) and modeled by the regression equation (solid line) at TN (**a**, **b**), TC (**c**, **d**), and NT (**e**–**g**) plantations in Taiwan

Daily soil respiration rates calculated using the regression model [Eq. (3)] are shown in Fig. 3. The trend between measured and regression model are well corresponding, and the daily soil respiration variation could be found in the regression model. The annual soil respiration rates that summed by daily soil respiration rates were estimated as 17.6 and 20.0 Mg C ha^−1^ year^−1^ at TN1 and TN2, 14.3 and 17.3 Mg C ha^−1^ year^−1^ at TC1 and TC2, and 7.6, 7.0, and 12.2 Mg C ha^−1^ year^−1^ at NT1, NT2, and NT3, respectively (Table 3). Although variations were observed on the stand scale, higher annual soil respiration rates were observed at the lowland TC and TN plantations than at the mid-elevation NT plantation.

**Table 3:** Estimated mean soil respiration rate and annual soil respiration rate in 2010 at TN, TC, and NT plantations in Taiwan

	Mean soil respiration rate (μmol CO_2_ m^−2^ s^−1^)	Annual soil respiration rate (Mg C ha^−1^ year^−1^)
TN1	4.67	17.6
TN2	5.30	20.0
TC1	3.78	14.3
TC2	4.58	17.3
NT1	2.02	7.6
NT2	1.86	7.0
NT3	3.22	12.2

**Fig. 4:**
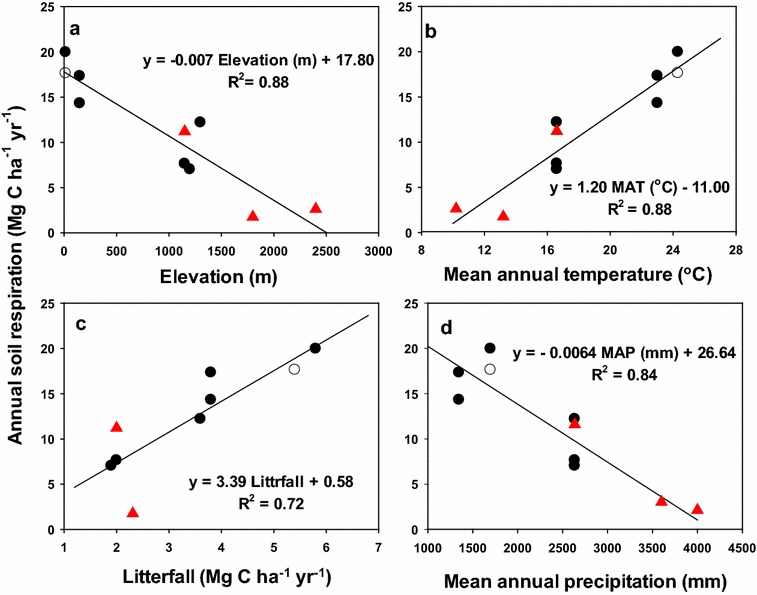
Linear relationships of annual soil respiration with **a** elevation, **b** mean annual temperature, **c** litterfall, and **d** mean annual precipitation in Taiwan. Black circles refer to the current study and red circles refer to previous studies (Chang et al. 2008; Kao and Chang 2009; Hsieh et al. 2016)

When combined with the finding of previous studies (Chang et al. 2008; Kao and Chang 2009; Hsieh et al. 2016), the annual soil respiration rates were strongly correlated with elevation, MAT, MAP, and litterfall production (p < 0.01, Table 4), explaining 88, 88, 84, and 72% of the variation in annual soil respiration, respectively (Fig. 4). Notably, the annual soil respiration rates increased by 1.2 Mg C ha^−1^ year^−1^ for every 1 °C increase in MAT and by 3.4 Mg C ha^−1^ year^−1^ for every 1-Mg C ha^−1^ year^−1^ increase in litterfall production. By contrast, the annual soil respiration decreased by 0.7 Mg C ha^−1^ year^−1^ for every 100-m increase in elevation and by 0.64 Mg C ha^−1^ year^−1^ for every 100-mm increase in MAP. Thus, higher annual soil respiration rates were found at the lowland plantations because of their higher MAT and litterfall production but lower MAP (< 1700 mm). By contrast, lower annual soil respiration rates were found at the plantations at higher elevations because of their lower MAT and litterfall production but higher MAP (> 2500 mm).

**Table 4:** Correlation coefficients at significant level p < 0.01 for annual soil respiration rate, elevation, mean annual temperature (MAT), mean annual precipitation (MAP), and litterfall production in 10 Taiwanese study stands

	Soil respiration (Mg C ha^−1^ year^−1^)	Elevation (m)	MAT (°C)	MAP (mm)	Litterfall (Mg C ha^−1^ year^−1^)
Soil respiration	1.00				
Elevation	− 0.93	1.00			
MAT	0.94	− 1.00	1.00		
MAP	− 0.92	0.94	− 0.94	1.00	
Litterfall	0.85	− 0.81	0.85	− 0.65	1.00

## Discussion

### Effects of soil temperature and soil water content on soil respiration

Soil temperature and soil water content are widely recognized as factors that control seasonal patterns of soil respiration (Raich and Schlesinger 1992; Saiz et al. 2006; Tang et al. 2006; Reichstein et al. 2003; Bond-Lamberty and Thomson 2010). In our study, we found that soil temperature or soil water content alone could somewhat account for the temporal pattern of soil respiration, and that the inclusion of both factors improved the predictive power of the model. The results suggested that the effects of the factors are interactive, rather than individual, for controlling the seasonal pattern of soil respiration. Generally, a higher soil respiration rate was observed in the hot and humid summer, whereas a lower soil respiration rate was observed in the cold and dry season.

Soil temperature and soil water content affected soil respiration differently. Soil temperature was positively correlated with the soil respiration rate at all three plantations. Soil water content was positively correlated with soil respiration at the lowland plantations but was negatively correlated at the mid-elevation plantation. We interpreted this finding as follows: soil water content at the lowland plantations was insufficiently high to reach the oxygen limitation for impeding soil respiration, whereas soil respiration at the mid-elevation plantation was inhibited by abundant precipitation (Davidson et al. 1998). Chang et al. (2008) and Hsieh et al. (2016) have reported the similar results of decreasing soil respiration with increasing soil water content in mountainous areas in Taiwan. Such a bidirectional relationship between soil water content and soil respiration has been previously observed on the watershed scale with a complex terrain or hydrological drainage (Riveros-Iregui et al. 2012; Knowles et al. 2015; Berryman et al. 2015). Our study provides the first evidence of a bidirectional relationship over an elevation gradient caused by different precipitation patterns.

The estimated Q_10_ values ranging between 1.44 and 3.31 were consistent with the global median value of 2.4 (Raich and Schlesinger 1992). The lower Q_10_ values at NT were possibly due to the oxygen limitation during summer, which rendered soil respiration less responsive to temperature (Davidson et al. 1998). When soil water content was added to the model, the Q_10_ values at NT increased from 1.44–2.37 to 1.92–3.60. The Q_10_ values at NT derived from the soil temperature and soil water content model [Eq. (3)] may be more accurate in representing the temperature dependence of soil respiration because the model considered the confounding effects of soil water content. However, the Q_10_ values at TN and TC derived from the combined soil temperature/soil water content model decreased from 2.44–3.31 to 1.39–2.69. The reduced Q_10_ values may have been masked by the correlation between soil temperature and soil water content (Tang et al. 2006).

### Annual soil respiration rates

The average annual soil respiration rates at TN, TC, and NT were estimated as 19, 16, and 10 Mg C ha^−1^ year^−1^, respectively. The annual soil respiration rates at TN and TC are consistent with those reported for other tropical forests such as the Amazon rainforest and those in Thailand, Malaysia, and Hawaii (Davidson et al. 2000; Hashimoto et al. 2004; Katayama et al. 2009; Litton et al. 2011). This finding indicated that both plantations are highly productive. In fact, the annual soil respiration rates at TN and TC are high compared with the rates reported by numerous studies worldwide (Raich and Schlesinger 1992; Bond-Lamberty and Thomson 2010). This result indicated that the rates at these plantations are significant to global soil respiration models. The annual soil respiration rate at NT is high compared with those recorded at Japanese cedar plantations in Japan (Shutou and Nakane 2004; Lee et al. 2006), possibly because of the higher annual temperature and active plant growth rates at NT (Cheng et al. 2013b).

In the present study, tight linkages were observed among annual soil respiration, elevation, MAT, MAP, and litterfall production at Taiwanese forest plantations (Table 4; Fig. 4). These results were expected because both abiotic (temperature and precipitation) and biotic (litterfall production) factors are critical drivers of soil respiration (Raich and Schlesinger 1992; Reichstein et al. 2003; Bond-Lamberty and Thomson 2010). The MAT, MAP, and litterfall production at all plantations were correlated with elevation (Fig. 4). Thus, elevation can be considered the main determinant factor influencing MAT, MAP, and primary production and defining the magnitude of the soil respiration rate. Previous studies have similarly reported that elevation is a crucial factor controlling the soil respiration rate (Kane et al. 2003; Litton et al. 2011; Berryman et al. 2014). Based on Fig. 4a, the annual soil respiration rate decreased by 0.7 Mg C ha^−1^ for every 100-m increase in elevation. However, such a relationship may be modified if the findings of new studies are added. For example, Raich and Tufekcioglu (2000) suggested that soil respiration rates in coniferous forests are 10% lower than those in broad-leaved forests. Broad-leaved forests in mountainous areas were not included in the present study.

In addition to climatic influence, differences in stand characteristics such as species composition, stand production, soil properties, and disturbances contribute to the variation in soil respiration (Raich and Tufekcioglu 2000; Tang et al. 2006; Bond-Lamberty and Thomson 2010; Sheng et al. 2010). In the present study, higher soil respiration rates at TN2 and NT3 might be because of their larger litterfall production and active plant growth rates (Table 1) (Lin et al. 2011; Cheng et al. 2013b). The higher soil respiration rate at TC2 was likely due to the invasive grass that accelerated the turnover rate of soil organic matter (Liao et al. 2008; Ward et al. 2009).

### Conceptual model for soil respiration in Taiwan

A conceptual model of biotic and abiotic factors affecting soil respiration was developed to improve the understanding of soil respiration in Taiwan. We proposed three determinant factors influencing soil respiration patterns and rates: (i) elevation, (ii) stand characteristics, and (iii) soil temperature and soil moisture.

(i) *Elevation* On the interregional scale, elevation significantly affected MAT, MAP, and primary production and thus defined the magnitude of the soil respiration rate. Higher annual soil respiration rates were found at lowland plantations, whereas lower annual soil respiration rates were found at the plantations at higher elevations.

(ii) *Stand characteristics* On the stand scale, differences in stand characteristics exerted further influence on the soil respiration rate. In this study, larger litterfall production, active plant growth rates, and invasive grass tended to enhance soil respiration rates.

(iii) *Soil temperature and soil water content* On the temporal scale, soil respiration rates were found to be driven by the seasonality of soil temperature and soil water content. The soil respiration rates were higher in the hot and humid season than in the cold and dry season. The combined soil temperature and soil water content model indicated that soil temperature positively contributed to the soil respiration rate. In addition, a bidirectional relationship was observed between soil respiration and soil water content. At the lowland plantations, higher soil water content led to higher soil respiration, whereas excessive amounts of water at the mid-elevation plantation limited the oxygen supply, yielding lower soil respiration.

Based on the conceptual model, we anticipate that climate change will play a significant role in the responses of soil respiration to the determinant factors. In the scenario of global warming, rising temperatures can induce higher soil respiration. Changes in precipitation patterns may also affect soil respiration. Drying at mid-elevation might lead to the release of more soil organic carbon into the atmosphere; therefore, causing a shift from a carbon sink to a carbon source. Because of the high variability of soil respiration, more studies and data syntheses are required to accurately predict soil respiration in Taiwanese forests.

## Conclusions

Soil temperature and soil water content are widely recognized as factors that control the seasonal patterns of soil respiration. The study results suggested that the effects of soil temperature and soil water content are interactive, rather than individual, for controlling the seasonal pattern of soil respiration. A combined soil temperature and soil water model explained 54–80% of the seasonal variations. However, these two factors affected soil respiration differently. Soil temperature positively contributed to soil respiration at all three plantations, and a bidirectional relationship between soil respiration and soil water content was observed among different plantations. Higher soil moisture content at TN and TC resulted in higher soil respiration rates; however, higher soil water content at NT led to adverse effects. The average annual soil respiration rates at TN, TC, and NT were estimated as 19, 16, and 10 Mg C ha^−1^ year^−1^, respectively. When assembled with the findings of previous studies, tight linkages were observed among annual soil respiration, elevation, MAT, MAP, and litterfall production. Higher annual soil respiration rates were found at the lowland plantations because of their higher MAT and litterfall production but lower MAP. By contrast, lower annual soil respiration rates were found at the plantations at higher elevations because of their lower MAT and litterfall production and higher MAP. We proposed three determinant factors affecting soil respiration patterns and rates: (i) elevation, (ii) stand characteristics, and (iii) soil temperature and soil moisture. Based on the conceptual model, we anticipate that climate change will play a significant role in the future responses of soil respiration to the aforementioned factors. Because of the high variability of soil respiration, more studies and data syntheses are required to accurately predict soil respiration in Taiwanese forests.
