# Correction to: Soil respiration patterns and rates at three Taiwanese forest plantations: dependence on elevation, temperature, precipitation, and litterfall

**DOI:** 10.1186/s40529-017-0215-5

**Published:** 2017-12-12

**Authors:** Yu-Hsuan Huang, Chih-Yu Hung, I-Rhy Lin, Tomonori Kume, Oleg V. Menyailo, Chih-Hsin Cheng

**Affiliations:** 1 School of Forestry and Resource Conservation, National Taiwan University, Taipei, 106 Taiwan; 2 Institute of Forest Research RAI SR, Krasnoyarsk, 660036 Russia

## Correction to: Bot Stud (2017) 58:49 10.1186/s40529-017-0205-7

Unfortunately, the original article (Huang et al. 2017) contained some errors. Figure 4 displayed incorrectly. The correct Fig. 4 can be found below:

**Fig. 4:**
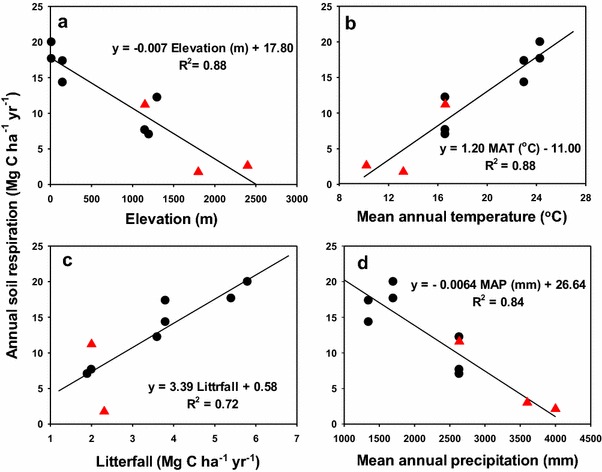
Linear relationships of annual soil respiration with **a** elevation, **b** mean annual temperature, **c** litterfall, and **d** mean annual precipitation in Taiwan. Black circles refer to the current study and red triangles refer to previous studies (Chang et al. 2001; Kuo and Chang 2009; Hsieh et al. 2016)
